# Spheroid Coculture of Human Gingiva-Derived Progenitor Cells With Endothelial Cells in Modified Platelet Lysate Hydrogels

**DOI:** 10.3389/fbioe.2021.739225

**Published:** 2021-08-26

**Authors:** Siddharth Shanbhag, Ahmad Rashad, Ellen Helgeland Nymark, Salwa Suliman, Catharina de Lange Davies, Andreas Stavropoulos, Anne Isine Bolstad, Kamal Mustafa

**Affiliations:** ^1^ Center for Translational Oral Research (TOR), Department of Clinical Dentistry, Faculty of Medicine, University of Bergen, Bergen, Norway; ^2^ Department of Immunology and Transfusion Medicine, Haukeland University Hospital, Bergen, Norway; ^3^ Department of Physics, Norwegian University of Science and Technology, Trondheim, Norway; ^4^ Department of Periodontology, Faculty of Odontology, Malmö University, Malmö, Sweden; ^5^ Division of Regenerative Medicine and Periodontology, University Clinics of Dental Medicine, University of Geneva, Geneva, Switzerland

**Keywords:** spheroid culture, coculture (co-culture), angiogenesis, bone tissue engineering, platelet lysate

## Abstract

Cell coculture strategies can promote angiogenesis within tissue engineering constructs. This study aimed to test the angiogenic potential of human umbilical vein endothelial cells (HUVEC) cocultured with gingiva-derived progenitor cells (GPC) as spheroids in a xeno-free environment. Human platelet lysate (HPL) was used as a cell culture supplement and as a hydrogel matrix (HPLG) for spheroid encapsulation. HUVEC and HUVEC + GPC (1:1 or 5:1) spheroids were encapsulated in various HPLG formulations. Angiogenesis was assessed via *in vitro* sprouting and *in vivo* chick chorioallantoic membrane (CAM) assays. HUVEC revealed characteristic *in vitro* sprouting in HPL/HPLG and this was significantly enhanced in cocultures with GPC (*p* < 0.05). A trend for greater sprouting was observed in 5:1 vs 1:1 HUVEC + GPC spheroids and in certain HPLG formulations (*p* > 0.05). Both HUVEC and HUVEC + GPC spheroids in HPLG revealed abundant and comparable neoangiogenesis in the CAM assay (*p* > 0.05). Spheroid coculture of HUVEC + GPC in HPLG represents a promising strategy to promote angiogenesis.

## Introduction

In the context of bone tissue engineering (BTE), timely vascularization of *in vivo* implanted constructs is critical for cell survival, especially in regions distant from the host vasculature, since diffusion of oxygen and nutrients is only limited to a distance of 150–200 µm (Jain et al., 2005; Nguyen et al., 2012). Angiogenesis is an essential component of the bone regeneration cascade and its insufficiency is a major limiting factor for the clinical translation of BTE strategies (Kanczler and Oreffo, 2008). Mesenchymal stromal cells (MSC) are increasingly being used for BTE (Pittenger et al., 2019; Shanbhag et al., 2019), and one strategy has been to coculture MSC with endothelial cells (EC), to create *in vitro* “pre-vascularized” constructs with a network of primitive vessels that functionally anastomose with the host vasculature when implanted *in vivo* (Levenberg et al., 2005; Rouwkema et al., 2006). MSC are reported to stabilize these networks by adopting a pericyte-like phenotype, thereby enhancing EC-mediated angiogenesis and in turn, bone regeneration (Keramaris et al., 2012; Loibl et al., 2014; Shanbhag et al., 2017a).

MSC derived from bone marrow (BMSC) are the most widely tested. However, progenitor cells from less-invasive sources, e.g., adipose and oral tissues, are being explored (Friedenstein et al., 1968; Pittenger et al., 2019). Oral tissues, such as dental pulp, periodontal ligament and gingiva, represent alternative sources of “MSC-like” progenitor cells (Sharpe, 2016). Gingiva, in particular, can be harvested with minimal morbidity and contains a subpopulation of multipotent progenitor cells (GPC), which demonstrate an MSC-like phenotype, immunomodulatory properties, and osteogenic potential both *in vitro* and *in vivo* (Fournier et al., 2010; Mitrano et al., 2010), thus representing promising alternatives to BMSC for BTE applications (Stefanska et al., 2020).

A critical aspect in the clinical translation of cell therapies is the use of safe and standardized culture conditions. Although commonly used for MSC expansion, several limitations of xenogeneic fetal bovine serum (FBS) supplementation have been highlighted, and current recommendations from health authorities advocate the use of “xeno-free” protocols whenever possible (Bieback et al., 2019a). Accordingly, xeno-free alternatives such as pooled human platelet lysate (HPL), have emerged and have been shown to be comparable, and often superior, to FBS for the proliferation and differentiation of various types of MSC (Fekete et al., 2012; Shanbhag et al., 2017b; Shanbhag et al., 2020a). We have recently reported that xeno-free culture of human GPC in HPL vs FBS media results in enhanced growth, gene expression and differentiation (Shanbhag et al., 2020b). Moreover, the proliferation and tube formation of EC is reported to be enhanced in HPL (Tasev et al., 2015) and other xeno-free media (Bauman et al., 2018).

Current BTE strategies rely mainly on monolayer expansion of MSC in plastic-adherent cultures (Rojewski et al., 2019). However, this two-dimensional (2D) culture system is not representative of the 3D *in vivo* microenvironment of MSC and may therefore alter their phenotype and diminish their regenerative and immunomodulatory potential (Banfi et al., 2000; Hoch and Leach, 2015; Ghazanfari et al., 2017). Similar observations have been reported in EC; single dissociated EC are reported to be more likely to undergo apoptosis (Korff and Augustin, 1998). In contrast, the self-assembly or spontaneous aggregation of cells into 3D spheroids is mediated by unique cell-cell and cell-extracellular matrix (ECM) interactions, biomechanical cues and signaling pathways, which more closely simulate the *in vivo* microenvironment (Sart et al., 2014; Cesarz and Tamama, 2016). In contrast to 2D monolayers, 3D spheroid culture has been reported to enhance survival, stemness, paracrine activity, immunomodulation and multi-lineage differentiation of MSC (Follin et al., 2016; Petrenko et al., 2017) (Kale et al., 2000; Chatterjea et al., 2017). In the context of BTE applications, we have observed particular advantages of spheroid vs monolayer culture via a strong upregulation of osteogenesis-related genes in BMSC and GPC (Shanbhag et al., 2020b).

Traditional cell delivery methods involve direct seeding and attachment of cells on biomaterial scaffolds before *in vivo* transplantation. However, direct seeding may not be the optimal method for delivery of cell spheroids because the 3D structure, essential to maximize their *in vivo* effects, is lost. To preserve the 3D structure, encapsulation of spheroids in hydrogels represents an effective delivery system (Murphy et al., 2014; Murphy et al., 2015; Ho et al., 2018). Moreover, in the context of angiogenesis, when EC are cultured as spheroids in a hydrogel matrix, either alone or in coculture with MSC, 3D network formation occurs by closely mimicking *in vivo* sprouting angiogenesis (Korff and Augustin, 1999; Heiss et al., 2015). Since HPL is increasingly being used for clinical-grade MSC culture (Bieback et al., 2019b), extending its application as a hydrogel carrier represents a cost-effective strategy for tissue engineering. Furthermore, HPL gels may offer the added advantage of sustained cytokine release at regeneration sites (Robinson et al., 2016). Indeed, recent studies have demonstrated the potential of HPL hydrogels for encapsulating EC and MSC to create microvascular networks (Fortunato et al., 2016; Robinson et al., 2016).

Previous studies have investigated the capacity of MSC to support or enhance EC-mediated angiogenesis in monolayer cultures, most often in xenogeneic conditions (Steffens et al., 2009; Verseijden et al., 2010; Chen et al., 2013; Ucuzian et al., 2013; Strassburg et al., 2016). Others have studied angiogenesis-related outcomes in spheroid cocultures of MSC or fibroblasts with EC in xeno-free, i.e., human serum-supplemented, media (Eckermann et al., 2011; Bauman et al., 2018). In the former study, MSC-EC cocultures in xeno-free media (vs FBS) resulted in enhanced angiogenesis in an *in vivo* chick chorioallantoic membrane (CAM) assay. With this background, the objective of the present study was to test the *in vitro* and *in vivo* angiogenic potential of EC cocultured with GPC as 3D spheroids encapsulated in HPL hydrogels.

## Materials and Methods

### Cell Culture

The use of human cells and tissues was approved by the Regional Committees for Medical Research Ethics (REK) in Norway (2016–1266, REK sør-øst C). Monolayer cultures of primary human GPC isolated from healthy donors were established in 5% HPL (Bergenlys®, Bergen, Norway). Details of isolation and characterization of GPC have been reported elsewhere (Shanbhag et al., 2020b). Early passage human umbilical vein EC (HUVEC) were purchased and cultured in EGM-2 growth medium (both from Lonza Inc., Walkersville, United States) supplemented with either 2% FBS, as per the manufacturer’s recommendations, or with 5% HPL; all other media components were maintained. Cells were sub-cultured and expanded under humidified 5% CO_2_ at 37°C; passages 2-4 were used in experiments. Functionality of HPL cultured HUVEC was tested in an *in vitro* tube formation assay on matrigel (Corning, NY, United States), as previously described (Fujio et al., 2017). Phase contrast images (Nikon Eclipse TS100, Tokyo, Japan) were analyzed using ImageJ software (NIH, Bethesda, United States) and angiogenesis-related parameters (tube length, branching, segments and junctions) were automatically quantified using the Angiogenesis Analyzer plugin, as previously described (Carpentier et al., 2020).

### 3D Spheroid (co)Culture

3D aggregate spheroids of HUVEC were formed via guided self-assembly in microwell plates as recently described (Shanbhag et al., 2020b). Briefly, suspensions of dissociated monolayer HUVEC cultured in FBS or HPL, were seeded in microwell plates (Sphericalplate®, Kugelmeiers Ltd., Erlenbach, Switzerland) for 24 h to form spheroids of ∼1000 cells each. Cell viability in spheroids was assessed via the LIVE/DEAD® kit (Invitrogen). Sprout formation in FBS and HPL cultured HUVEC spheroids was assessed using phase and confocal microscopy: for the latter, immunofluorescence (IF) staining with CD31 was performed (Supplementary methods). For subsequent experiments, all cell culture was performed in HPL media. For coculture spheroids, microwells were seeded with suspensions of dissociated HUVEC and GPC in two different ratios, 1:1 and 5:1 (HUVEC:GPC), based on previous work (Ma et al., 2011; Pedersen et al., 2013). After 24 h, HUVEC and HUVEC-GPC spheroids were collected by gentle pipetting and encapsulated in HPL hydrogels (HPLG).

### Encapsulation in Hydrogels

Since HPL was used to establish xeno-free cultures of GPC and HUVEC, its application as a hydrogel scaffold was also investigated. Initially, HPLG were produced via addition of thrombin solution [1 IU/ml human thrombin and one TIU/ml aprotinin in 40 mM CaCl_2_ solution (all from Sigma-Aldrich)] to sterile-filtered HPL followed by incubation at 37°C for 15 min. The resulting hydrogel was referred to as “unmodified” HPLG (0F). For encapsulation, HUVEC or coculture spheroids were suspended in HPL solution, quickly mixed with the thrombin solution, and added to culture plates with gentle shaking to ensure uniform distribution of the spheroids. The plates were transferred to the incubator for 15 min to ensure complete gelation and thereafter supplemented with EGM-2 growth medium for the indicated culture periods.

Subsequently, to improve the hydrogels mechanical properties, HPL was supplemented with fibrinogen (Sigma-Aldrich) in concentrations of 1.25 (1.25F), 2.5 (2.5F), 6.25, 12.5, and 25 mg/ml. Gelation and spheroid encapsulation was performed using the same thrombin solution as described above. These hydrogels were referred to as “modified” HPLG. Rheological properties of modified HPLG were assessed as described in the Supplementary methods. Only 0, 1.25 and 2.5F HPLG were used in subsequent experiments (see *Hydrogel Properties Influence HUVEC Sprouting*).

### Sprouting Angiogenesis Assay

The *in vitro* angiogenic potential of mono- and coculture spheroids was tested in a sprout assay, as previously described (Nakatsu and Hughes, 2008). Briefly, HUVEC or HUVEC-GPC spheroids were encapsulated in HPLG and cultured for 72 h in EGM-2 medium to observe sprout formation. In selective experiments, HUVEC spheroids (encapsulated in 0F HPLG) were cultured with a monolayer of GPC on top of the gel, i.e., “indirect” cocultures – to test whether paracrine factors from GPC influenced HIUVEC sprouting. In “direct” cocultures, prior to spheroid formation, dissociated GPC and HUVEC were labeled with fluorescent green (DiO, 5 μL/ml) and red (Dil, 5 μL/ml) dyes (Vybrant® cell-labelling solution, Invitrogen), respectively. HUVEC-only spheroids (only red-labelled cells) were formed as controls. Spheroids of HUVEC or HUVEC-GPC (1:1 or 5:1 HUVEC:GPC) were encapsulated in modified HPLG (0, 1.25 or 2.5F), and cultured in EGM-2 for up to 72 h in 8-well μ-slides® (ibidi, Munich, Germany). Subsequently, the constructs were fixed in 4% paraformaldehyde (PFA) and permeabilized using 0.2% Triton X-100 (Sigma-Aldrich). Prior to imaging, nuclei were stained using 4′,6-diamidino-2-phenylindole (DAPI, Sigma-Aldrich).

### Confocal Microscopy

Whole mount imaging of HPLG-encapsulated spheroids was performed using an Andor Dragonfly 5050 high-speed confocal microscope and Fusion software (both from Oxford Instruments, Abingdon, United Kingdom). Z-stacks were acquired from the top of each gel, with steps of 4 μm to a depth of up to 200 μm. Each image was captured with a high-speed iXon 888 Life EMCCD camera with 1024 × 1024 resolution at 100–200 × magnification. Green (GPC) and red (HUVEC) stained cells/sprouts, and their nuclei (DAPI), were scanned in the corresponding channels using 546, 647 and 405 lasers, respectively. Images were processed using the Imaris software (Oxford Instruments) and transferred to ImageJ (NIH) for analysis. Using the Sprout Morphology plugin, segmentation and thresholding of images was performed to separate GPC, HUVEC and nuclei. Images were calibrated using scale bars and sprout lengths (in μm) were automatically or manually calculated using ImageJ, as described elsewhere (Eglinger et al., 2017), on segmented images showing only HUVEC in the red-channel.

### CAM Assay

The *in vivo* angiogenic potential of mono- and coculture spheroids was tested in an *ex ovo* CAM assay in developing chick embryos, in accordance with the Norwegian Animal Research Authority (Mattilsynet), where an experimental period < 14 days did not require formal ethical approval. Briefly, fertilized chicken eggs were incubated at 37°C for 72 h with intermittent rotation. On embryonic day 3, the eggs were carefully opened, their contents transferred into petri dishes and incubated in humidified air at 37°C. On day 7, HUVEC or HUVEC-GPC spheroids encapsulated in 1.25F HPLG (50 spheroids in 50 µL gel; 1:1 HPL:EGM-2) were implanted on the CAMs avoiding pre-existing blood vessels. To maintain their positions on the CAMs, the gels were contained within silicone O-rings (⌀ 10 mm). During the incubation period, some embryos were terminated as a result of embryonic death unrelated to the implants. Implants from these terminated embryos were harvested for live cell-staining using Calcein AM (Invitrogen). On day 14, the regions within the O-rings in the remaining embryos were recorded using a digital stereomicroscope (Leica Biosystems, Heerbrugg, Switzerland). Subsequently, the CAMs were fixed in 4% PFA and regions around the O-rings were harvested, embedded in paraffin and analyzed histologically following hematoxylin and eosin staining. Quantification of angiogenesis-related parameters (vessel density, vessel length, segments and branching points) in CAM images was performed using the Wimasis® automated image analysis software (Onimagin Technologies, Cordoba, Spain) (Montali et al., 2017).

### Statistical Analysis

Statistical analysis was performed using the Prism 9.0 software (GraphPad Software, San Diego, CA, United States). Data are presented as means ± SD, unless specified. Normality testing was performed via the Shapiro-Wilk test. The student *t* test, Mann-Whitney U test and one-way analysis of variance (ANOVA), followed by post-hoc Tukey’s or Dunn’s test for multiple comparisons, were applied when appropriate and *p* < 0.05 was considered statistically significant.

## Results

### HPL Supports Xeno-free Culture of HUVEC

**FIGURE 1:**
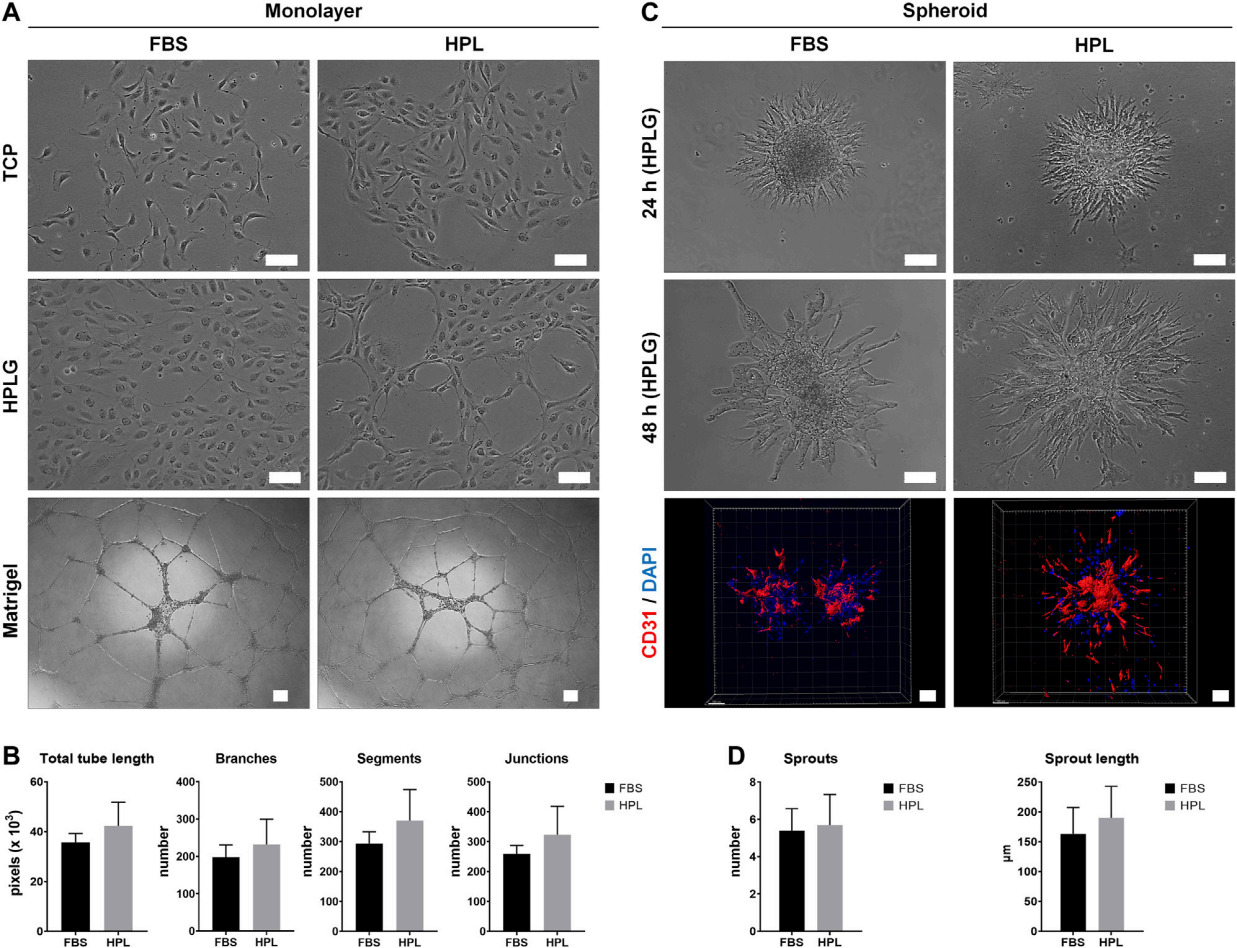
Xeno-free HUVEC culture in HPL. **(A)** Comparison of HUVEC morphology in HPL and FBS on tissue culture plastic (TCP) and HPLG, and corresponding tube formation on Matrigel after 24 h; scale bars 100 µm. **(B)** Analysis of tube formation parameters; data represent means ± SD (n = 3). **(C)** Comparison of *in vitro* sprouting by HUVEC spheroids in HPL and FBS after 24 and 48 h in HPLG; IF staining for CD31 (HUVEC, red) and DAPI (nuclei, blue) in 48 h-spheroids; scale bars 100 µm. **(D)** Analysis of sprout formation parameters; data represent means ± SD (n = 3 or more).

Monolayer HUVEC were successfully cultured by substituting 2% FBS with 5% HPL in EGM-2 media. When cultured on tissue culture plates coated with unmodified HPLG, spontaneous tube-like organization of HPL cultured HUVEC was observed (Figure 1A). In the matrigel assay, a trend for superior tube formation was observed in HPL vs FBS cultured HUVEC; quantification of all angiogenesis-related parameters revealed a higher trend in HPL, without significant differences (*p* > 0.05; Figure 1B). Spheroids of HUVEC in HPL and FBS media were formed and encapsulated in unmodified HPLG; high cell viability in the spheroids was observed after 48 h (data not shown). Sprout formation was initiated at 24 h and increased over time in both FBS and HPL cultured HUVEC; detection of CD31 in HUVEC sprouts was confirmed via IF and confocal microscopy (Figure 1C). A trend for increased sprouting (sprout numbers and length) was observed in HPL vs FBS cultured HUVEC spheroids, without significant differences (*p* > 0.05; Figure 1D).

### Spheroid Coculture Promotes Sprouting Angiogenesis

**FIGURE 2:**
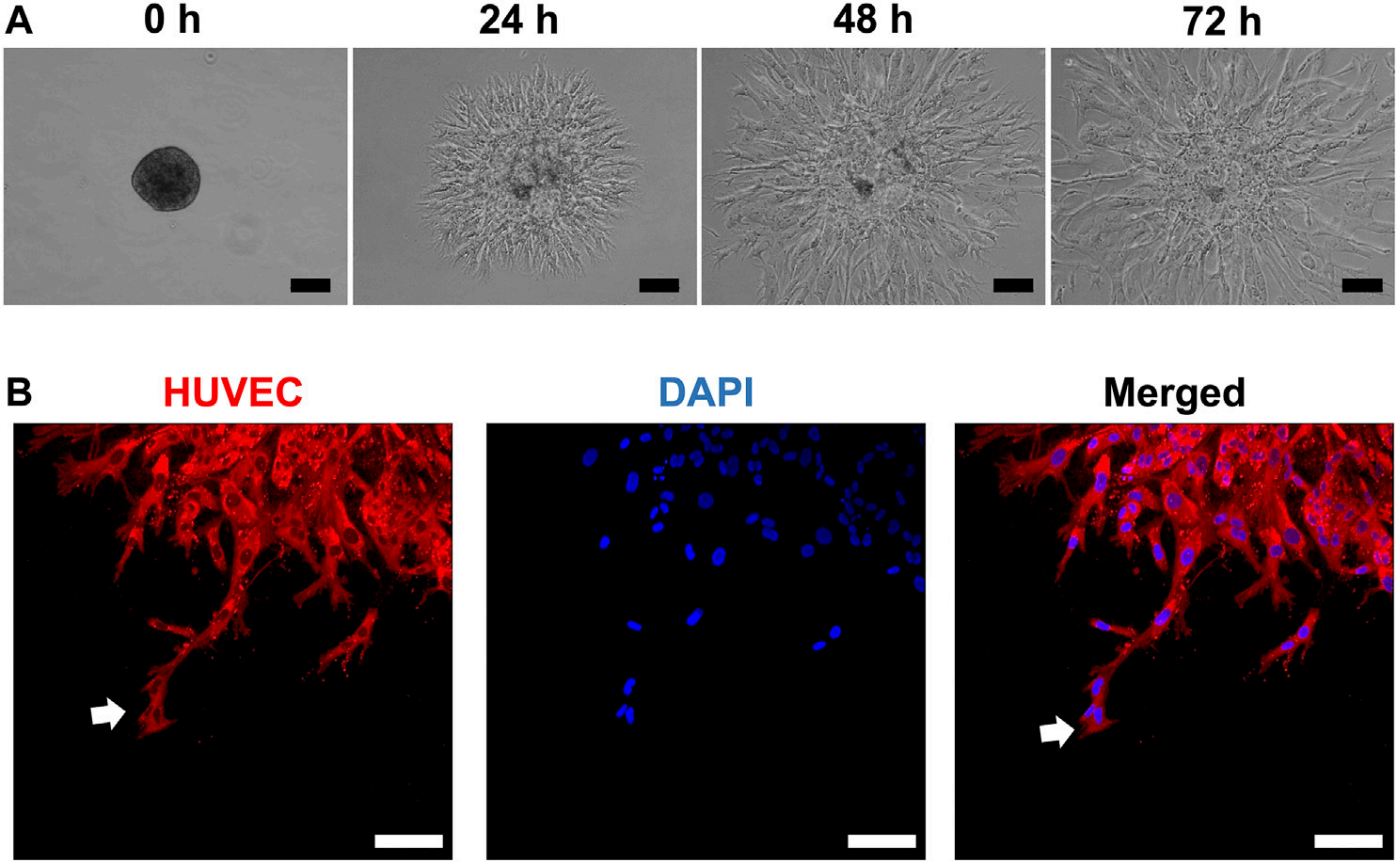
HUVEC sprouting in HPLG. **(A)** Representative phase-contrast images showing the progression of sprout formation in xeno-free HUVEC spheroids in HPLG; scale bars 100 µm. **(B)** Representative confocal images showing sprout formation by HUVEC monoculture spheroids in unmodified HPLG; initiation by sprouting by “tip cells” (white arrows) after 24 h; scale bars 100 µm.

HUVEC sprouting was assessed first in unmodified (and later in modified) HPLG. Generally, sprouts appeared as narrow tube-like structures after 24 h, guided by characteristic “tip” cells, extending from the spheroid surface into the gel matrix and progressively increasing in length (Figures 2A,B). After 72 h, abundant network formation was observed between the sprouts of adjacent spheroids. In “indirect” cocultures, i.e., when monolayer GPC were seeded on top of HPLG encapsulating HUVEC spheroids, a trend for increased sprouting was observed in HUVEC with vs without overlying GPC (*p* > 0.05; Supplementary Figure 1).

**FIGURE 3:**
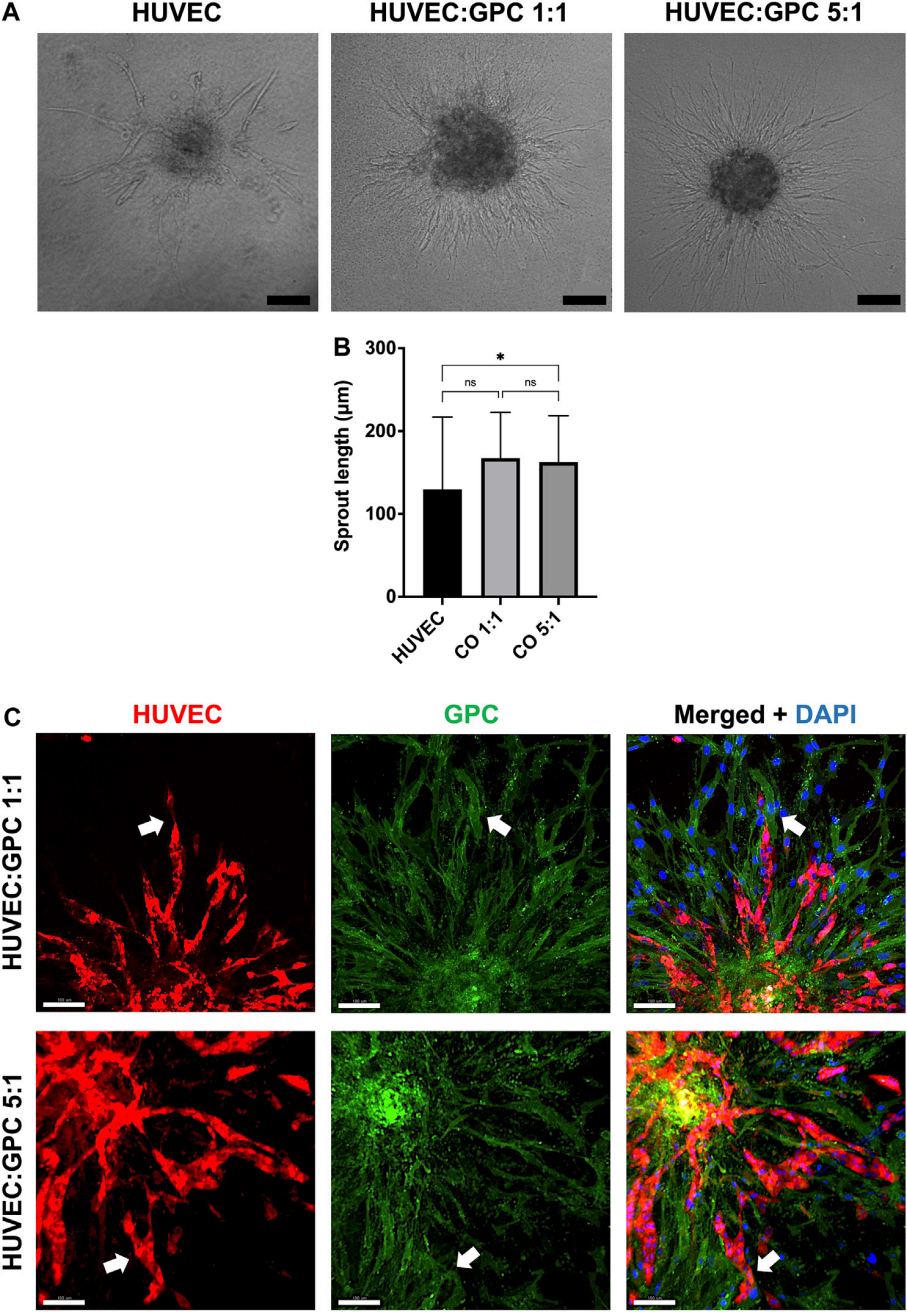
Coculture of GPC and EC in HPLG. **(A)** Representative images of HUVEC sprouting (and GPC spreading) in HUVEC monoculture and 1:1 and 5:1 (HUVEC:GPC) coculture spheroids after 72 h in unmodified HPLG (scale bars 100 μm). **(B)** Quantification of corresponding sprout lengths based on dual-staining and confocal imaging – only red-stained HUVEC sprouts were measured; *p < 0.05; data represent means ± SD of at least three experimental repeats (n ≥ 5 spheroids per experiment). **(C)** Representative confocal images showing sprout formation in 1:1 and 5:1 HUVEC:GPC coculture spheroids; white arrows indicate GPC (green) organization along HUVEC sprouts (red;); nuclei are stained with DAPI (scale bars 100 µm).

In “direct” coculture spheroids, sprout formation by HUVEC was accompanied by spreading/migration of GPC within HPLG (Figure 3A). Both HUVEC and GPC showed high viability (Supplementary figure 2A). When testing different coculture ratios, spheroids of 5:1 HUVEC:GPC revealed significant increases in sprout length vs HUVEC-only spheroids (*p* < 0.05; Figure 3B). No significant differences were observed between 1:1 and 5:1 coculture spheroids (*p* > 0.05). Dual cell-labelling revealed GPC to be organized along, and in direct contact with, HUVEC sprouts (Figure 3C). GPC spreading preceded HUVEC sprouting and appeared to provide a substrate for HUVEC migration and sprouting (Supplementary figure 2B).

### Hydrogel Properties Influence HUVEC Sprouting

**FIGURE 4:**
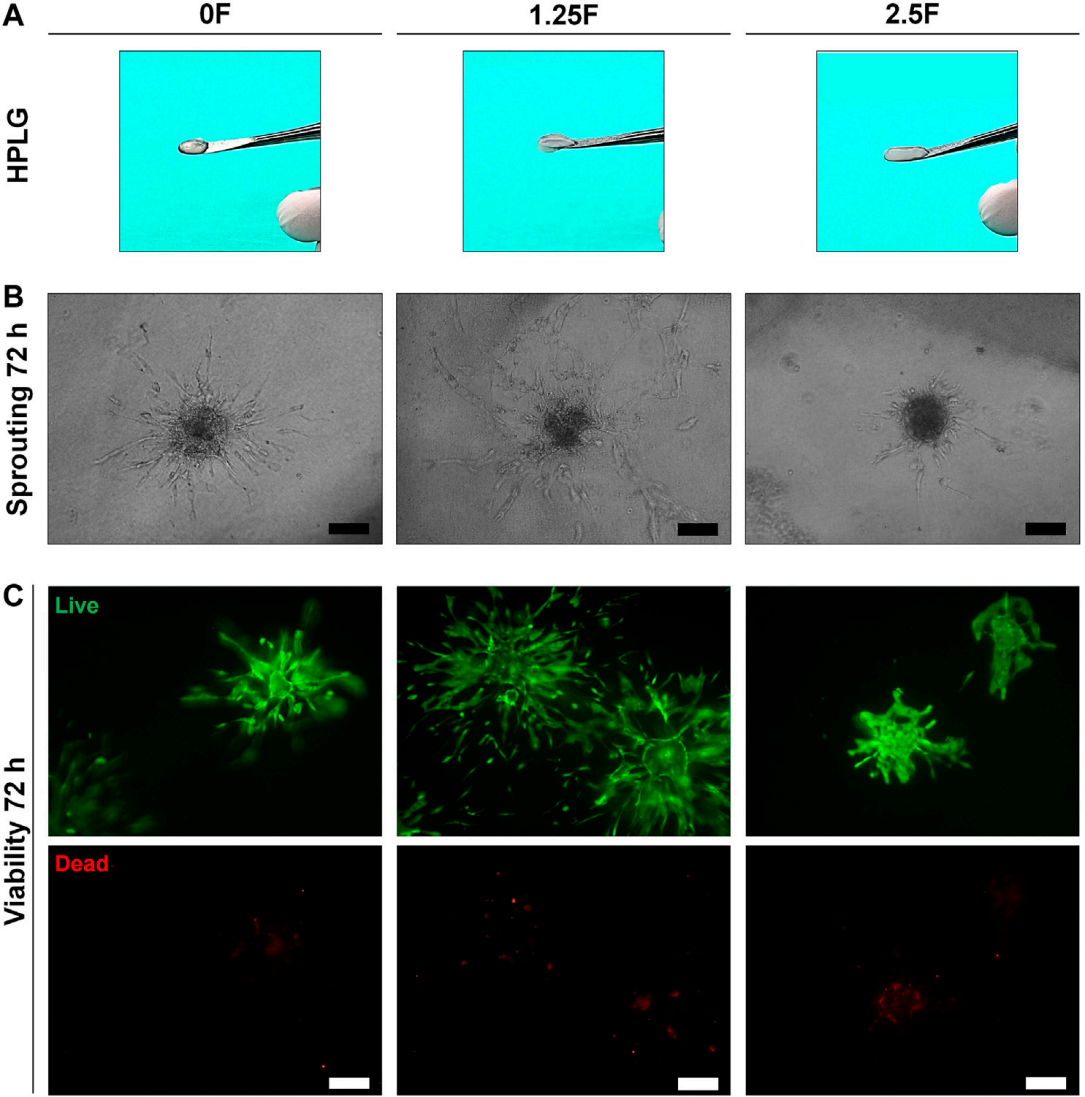
Optimization of HPLG. **(A)** Representative photographs of unmodified (0F) and modified HPLG supplemented with 1.25 (1.25F) or 2.5 mg/ml fibrinogen (2.5F). **(B)** Representative phase contrast images of HUVEC sprouting after 72 h in the corresponding HPLG (scale bars 100 μm). **(C)** Cell viability via LIVE/DEAD assay in HUVEC spheroids after 72 h in the corresponding HPLG (scale bars 100 μm).

**FIGURE 5:**
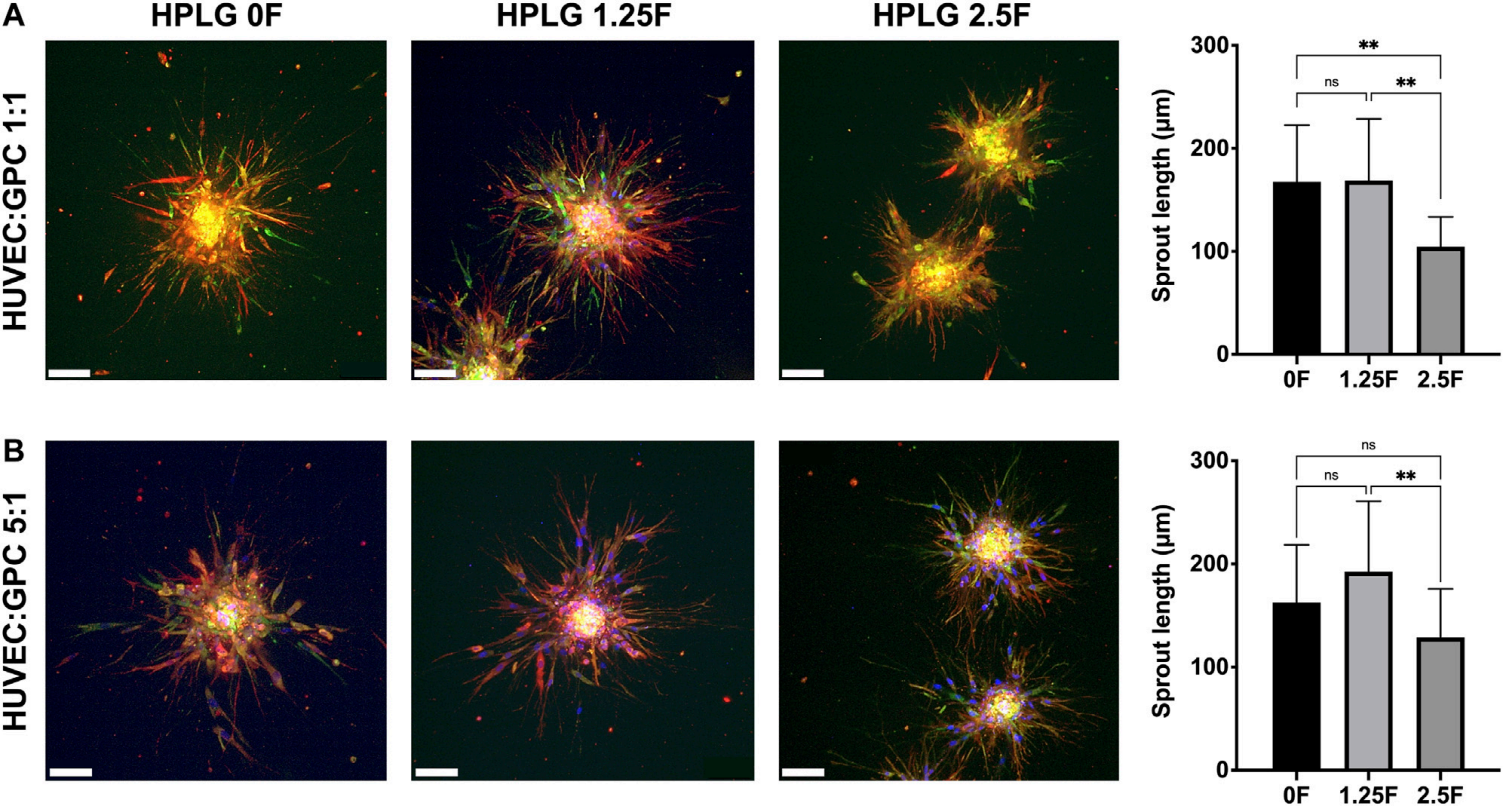
Optimization of coculture ratios. Representative confocal images of HUVEC-sprouting (red) and GPC-spreading (green) after 72 h in 1:1 **(A)** and 5:1 **(B)** HUVEC:GPC coculture spheroids in 0, 1.25 and 2.5F HPLG (scale bars 100 μm) and corresponding quantification of sprout lengths; **p < 0.001; data represent means ± SD of at least three experimental repeats (n = ≥ 5 spheroids per experiment).

Modified HPLG were produced by supplementing HPL with fibrinogen (Figure 4A). High cell viability and favorable sprouting of HUVEC spheroids were observed in HPLG with ≤ 2.5 mg/ml fibrinogen (Figures 4B,C). Spheroids in HPLG with >2.5 mg/ml fibrinogen showed no sprouting and many dead cells (Supplementary figure 4A–C). Therefore, only unmodified HPLG (0F) or 1.25 and 2.5F modified HPLG were used in subsequent experiments. Rheology revealed corresponding increases in storage and loss moduli of HPLG with increasing concentrations of fibrinogen (Supplementary figure 4). In 1:1 HUVEC:GPC cocultures, sprouting was comparable in 0F and 1.25F HPLG, and significantly greater than in 2.5F HPLG after 72 h (*p* < 0.05; Figure 5A). In 5:1 HUVEC:GPC cocultures, a non-significant trend for superior sprouting was observed in 1.25F HPLG (*p* > 0.05, Figure 5B). Thus, the combination of 5:1 HUVEC:GPC and 1.25F HPLG was considered optimal and used in the CAM assay.

### Spheroid Coculture Supports Angiogenesis *in vivo*

**FIGURE 6:**
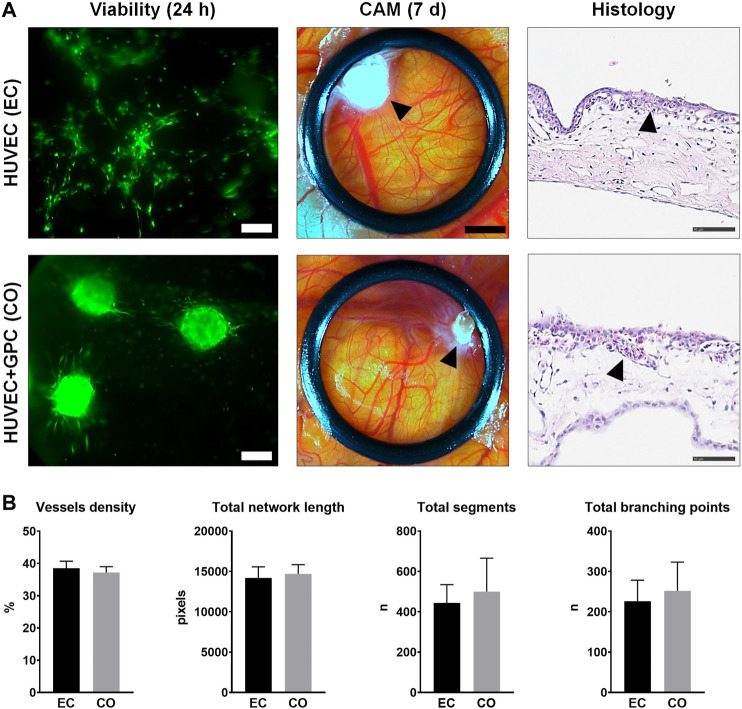
CAM angiogenesis assay. **(A)** Comparison of HUVEC (EC) and HUVEC-GPC coculture (CO) spheroids seeded in HPLG (L to R): cell viability after 24 h [green colour indicates viable cells, note the dissociation of HUVEC (EC) spheroids into tube-like networks; scale bars 100 μm], stereomicroscope images showing neoangiogenesis in CAMs after 7 days (black arrows indicate condensed HPLG within the O-rings; scale bars 200 μm) and corresponding histological images with H & E staining (black arrows indicate newly formed vessels; scale bars 50 μm). **(B)** Analysis of angiogenesis-related parameters based on stereomicroscope images; n = number; data represent means ± SD (n = 3).

HUVEC and HUVEC-GPC (5:1) spheroids in 1.25F HPLG were implanted on developing chicken embryo CAMs. *In vitro* sprout formation by the encapsulated spheroids was confirmed (Supplementary figure 5). Live cell-staining of gels harvested 24 h after implantation revealed high cell viability. While HUVEC spheroids appeared to dissociate and organize into networks, HUVEC-GPC spheroids retained their 3D structure and showed characteristic sprouting on the CAMs (Figure 6A). After 7 days of implantation, active angiogenesis with dense vascular networks was observed in the regions of both HUVEC and HUVEC-GPC implants. Although the spheroids were evenly distributed in the gels at the time of implantation, after 7 days they appeared to be aggregated to one side of the O-rings and the HPLG was almost completely degraded (Figure 6A). Histology revealed a high density of vessels at the CAM surface, to a similar degree in both groups (Figure 6A). Degradation of HPLG precluded the detection of construct integration via penetration of CAM vessels into the gels. Quantification of angiogenesis revealed no significant differences between HUVEC and HUVEC-GPC spheroids for any of the tested parameters (*p* > 0.05; Figure 6B).

## Discussion

3D cocultures of MSC and EC can promote angiogenesis and potentially overcome the challenges of *in vivo* vascularization in BTE constructs (Rouwkema et al., 2006; Nguyen et al., 2012). The aim of this study was to test whether GPC, as an alternative to BMSC, supported EC-mediated sprouting angiogenesis in xeno-free HPL cultures.

HUVEC represent a feasible and frequently used model to study EC behavior in experimental settings (Morin and Tranquillo, 2013). Consistent with previous reports, tube formation and sprouting by HUVEC was improved in HPL vs FBS. Sprouting angiogenesis by 3D-cultured EC *in vitro* is considered to be a close representation of the *in vivo* angiogenic cascade, recapitulating all the key events during which quiescent EC become activated to proteolytically degrade their surrounding ECM, e.g., hydrogels, directionally migrate towards the angiogenic stimulus, proliferate, and organize into new 3D capillary networks (Korff and Augustin, 1999; Chappell et al., 2012; Nowak-Sliwinska et al., 2018). Moreover, these sprout-networks have revealed functional lumenized capillaries, which anastomose with host vasculatures when implanted *in vivo* (Alajati et al., 2008; Finkenzeller et al., 2009; Morin and Tranquillo, 2011). A recent study reported superior sprouting of EC in human serum vs FBS supplemented media (Bauman et al., 2018). Consistently, a trend for superior sprout formation by HPL vs FBS cultured HUVEC spheroids was observed herein. Thus, HPL appears to be a feasible xeno-free alternative for HUVEC culture.

The formation and stability of *in vitro* and *in vivo* capillary-like networks by EC can be enhanced via coculture with MSC (Pedersen et al., 2012; Pedersen et al., 2013; Ma et al., 2014). We have previously reported that GPC demonstrate MSC-like phenotype and properties in xeno-free cultures (Shanbhag et al., 2020b). Accordingly, 3D cocultures of HUVEC and GPC were established herein. To test whether GPC promoted HUVEC sprouting via cell-to-cell contact or paracrine mechanisms, direct and indirect cocultures were established, respectively. While indirect coculture with GPC revealed a trend for greater HUVEC sprouting, direct coculture with GPC in a 5:1 ratio significantly improved HUVEC sprouting. These results are consistent with previous studies of HUVEC spheroids cocultured with BMSC (Hsu et al., 2014; Robinson et al., 2016; Bauman et al., 2018), and studies highlighting the importance of direct cell-to-cell contacts, rather than paracrine interactions, in coculture settings (Ball et al., 2004; Liang et al., 2017).

To optimize the 3D cocultures, two different coculture ratios were tested. While a 1:1 ratio of MSC and EC is most frequently reported (Ma et al., 2011; Shanbhag et al., 2017a), previous studies from our group and others have suggested that higher proportions of EC may improve angiogenesis in cocultures (Verseijden et al., 2010; Pedersen et al., 2012; Pedersen et al., 2013). However, no significant differences in HUVEC sprouting were observed between high (5:1) and low (1:1) coculture ratios herein. Notably, only the 5:1 cocultures showed significantly greater sprouting vs HUVEC only spheroids. Considerably greater spreading or migration of GPC was observed in spheroids with relatively more GPC, i.e., in 1:1 spheroids. Spreading preceded HUVEC sprouting and may have provided a substrate for sprout growth and elongation. Indeed, MSC are reported to show signs of pericytic differentiation, e.g., via expression of smooth-muscle markers, in EC cocultures (Lozito et al., 2009). Similar patterns of spreading by MSC have been reported in 3D cocultures embedded in collagen gels (Shah and Kang, 2018). This is in contrast to non-embedded 3D cocultures, where MSC do not spread, and EC, in the absence of an ECM, organize into internal networks within the spheroids rather than external sprouts (Rouwkema et al., 2006; Verseijden et al., 2010; Eckermann et al., 2011; Marshall et al., 2018). In the present study, an “embedded” spheroid model was selected to recapitulate angiogenic sprouting by using HPL hydrogels as ECM scaffolds to deliver the “pre-vascularized” constructs *in vivo* (Robinson et al., 2016).

Recent studies have demonstrated the benefits of HPLG for EC-mediated angiogenesis (Fortunato et al., 2016; Robinson et al., 2016). HPLG are produced by simulating the *in vivo* coagulation cascade, i.e., via addition of thrombin and/or CaCl_2_ to convert fibrinogen to fibrin, and thus represent highly biomimetic scaffolds for tissue engineering applications. Together with cell culture in HPL supplemented media, this would represent a fully xeno-free coculture system with a high potential for clinical translation. Although HPLG can support capillary-like network formation by EC, their mechanical properties may be considered insufficient for *in vivo* implantation, especially in non-contained bone defects. Thus, the HPLG were supplemented with fibrinogen for more predictable *in vivo* delivery. Fibrin gels are routinely used as scaffolds in a range of applications including BTE (Soffer et al., 2003). Moreover, fibrin gels have been extensively used to study EC sprouting angiogenesis (Morin and Tranquillo, 2013). Notably (unmodified) HPLG have been shown to be superior to fibrin gels in this regard (Robinson et al., 2016). However, the mechanical properties of unmodified HPLG may only allow injectable delivery due to their highly liquid nature. In the present study, it was hypothesized that supplementation of HPLG with fibrinogen would enhance the mechanical properties of the gels, while retaining the biological activity of HPL. Although the addition of fibrinogen seemingly improved the mechanical properties of HPLG, the biological activity (HUVEC viability and sprouting) declined beyond a concentration of 2.5 mg/ml. Interestingly, HUVEC sprouting in 1.25F gels was slightly enhanced vs unmodified HPLG and significantly enhanced vs 2.5F HPLG. This contrasted with a previous study comparing unmodified HPLG and 1.25 or 2.5 mg/ml fibrin gels (Robinson et al., 2016). In the context of BTE, hydrogel stiffness is also reported to influence MSC fate-determination and osteogenic differentiation (Hwang et al., 2015; Sun et al., 2018; Zigon-Branc et al., 2019). Our findings, together with previous reports (Rao et al., 2012; Hsu et al., 2014; Robinson et al., 2016), highlight the importance of ECM/scaffold properties on EC-mediated angiogenesis within tissue engineered constructs.

To test the *in vivo* angiogenic potential of spheroid-HPLG constructs, a CAM assay in the developing chick embryo was used. This offers a relatively rapid and cost-effective model for *in vivo* biomaterial/xenograft testing, particularly for angiogenesis, in a naturally immunocompromised host with a rapidly developing vascular bed (Moreno-Jimenez et al., 2016; Ribatti, 2016). Cell viability and sprouting of both HUVEC and HUVEC-GPC spheroids was confirmed after 24 h in excised CAMs. Interestingly, in the absence of GPC, HUVEC appeared to dissociate from spheroids and organize into tube-like networks as observed in monolayer cultures. Seven days after implantation, a dense network of capillaries was observed macroscopically in the CAM-regions implanted with both HUVEC and HUVEC-GPC spheroids. This is consistent with previous studies reporting angiogenesis in CAMs implanted with xenogeneic (Steffens et al., 2009; Strassburg et al., 2016) or xeno-free coculture spheroids (Bauman et al., 2018). In the latter study, the integration of sprouts with the CAM vasculature was confirmed via immunohistochemistry (Bauman et al., 2018). However, no significant advantage of HUVEC-GPC coculture was observed in the CAM assay herein, and therefore, the benefits of coculture observed for *in vitro* sprouting were not translated *in vivo*.

Some limitations of our study must be acknowledged. While most previous studies have reported the *in ovo* “eggshell window” method for the CAM assay (Steffens et al., 2009; Liu et al., 2012; Strassburg et al., 2016; Bauman et al., 2018), a complete *ex ovo* method was used in our study. In the former, the construct is placed on the CAM through an opening in the eggshell; retention of the embryo within the egg and coverage of the window during the experimental period are advantageous in terms of hydration and reduced risk of contamination. Exposure of the CAMs in our method contributed to dehydration and faster resorption of the HPLG, which may have compromised existing sprout-networks and precluded the formation of new sprouts. Moreover, a longer observation period was used herein (7 days) compared to previous reports (3 days) (Liu et al., 2012; Bauman et al., 2018), which may also have masked any “early differences” between the groups; a single “end-point” was selected herein to minimize disturbance and exposure of the CAMs. An *in ovo* model with shorter/multiple observation periods may offer a more reliable picture in future studies. Moreover, ectopic implantation of the constructs in more relevant animal models, e.g., immunocompromised mice, may provide further clues regarding hydrogel degradation and vascular anastomosis.

It has been reported that in the absence of supporting cells, EC networks are stable for a shorter duration *in vitro* (Pedersen et al., 2012; Pill et al., 2018). When implanted *in vivo*, the engineered vessels must remain stable long enough to anastomose with the native vasculature and sustain the implanted cells (Pedersen et al., 2013). In the present study, GPC were found to be organized in close contact with HUVEC sprouts and appeared to provide a “substrate” for sprout formation/elongation. Thus, it may be hypothesized that GPC could help to stabilize EC networks in more challenging *in vivo* conditions (Zhang et al., 2020). In the context of BTE, it is unclear whether MSC/GPC in cocultures serve dual functions of supporting angiogenesis and promoting osteogenesis, i.e., osteogenic differentiation and/or paracrine stimulation. In a meta-analysis of MSC-EC co-transplantation studies *in vivo*, we observed a significant benefit of coculture for bone, but not vessel, regeneration (Shanbhag et al., 2017a). Further research is needed to clarify whether MSC, and other supporting cells, adopt a pericyte- and/or osteoblast-like phenotype when cocultured with EC. Finally, further optimization of culture conditions, e.g., cell ratios, media, ECM/scaffolds, etc., to promote both osteogenesis and angiogenesis, and not one or the other, is needed prior to clinical application.

## Conclusion

In summary, HPL represents a suitable xeno-free alternative for HUVEC culture. HUVEC spheroids in HPL/HPLG demonstrated *in vitro* sprouting angiogenesis, which was significantly enhanced via direct coculture with GPC. A 5:1 HUVEC:GPC ratio in a specific HPLG formulation appeared to be optimal in terms of *in vitro* sprouting. Further optimizations of coculture conditions are needed to translate these *in vitro* findings in the appropriate *in vivo* models.

## Data Availability

The raw data supporting the conclusions of this article will be made available by the authors, without undue reservation.
