# Supplementary material for: Spheroid Coculture of Human Gingiva-Derived Progenitor Cells With Endothelial Cells in Modified Platelet Lysate Hydrogels
Source: Front Bioeng Biotechnol. 2021 Aug 26;9:739225. doi: 10.3389/fbioe.2021.739225 (PMC8427051; doi:10.3389/fbioe.2021.739225)
Supplement: Supplementary file 1 [file DataSheet1.docx]

**Spheroid coculture of human gingiva-derived progenitor cells with endothelial cells in modified platelet lysate hydrogels**

**Supplementary data**

1. **Supplementary methods**
   1. ***Immunofluorescence***

Expression of the endothelial marker CD31 was assessed in HUVEC sprouts via immunofluorescence. Briefly, HUVEC spheroids/sprouts in HPLG were fixed using 4% PFA for 20 min at RT, permeabilized with 0.2% Triton X-100 (Sigma-Aldrich), and incubated ON at 4°C with a mouse anti-human CD31 antibody (clone WM59; BD Pharmingen, Franklin Lakes, NJ, USA). Secondary antibody incubation was performed for 1 h at RT using a goat anti-mouse Alexa Fluor 635 antibody (Invitrogen). Nuclear staining was performed using 4′,6-diamidino-2-phenylindole (DAPI, Sigma-Aldrich) prior to imaging on a confocal microscope, as described in the main manuscript.

- 1. ***Rheology***

Rheological properties of 0F, 1.25F and 2.5F HPLG were assessed using a Physica MCR 301 rheometer (Anton Paar, Hertford Herts, UK). Frequency sweeps from 0.01 to 100 Hz at 37°C and 0.1% strain were performed and the storage modulus and loss modulus of the individual gels were obtained.

1. **Supplementary figures**
   1. **Supplementary figure 1: Indirect cocultures**

**
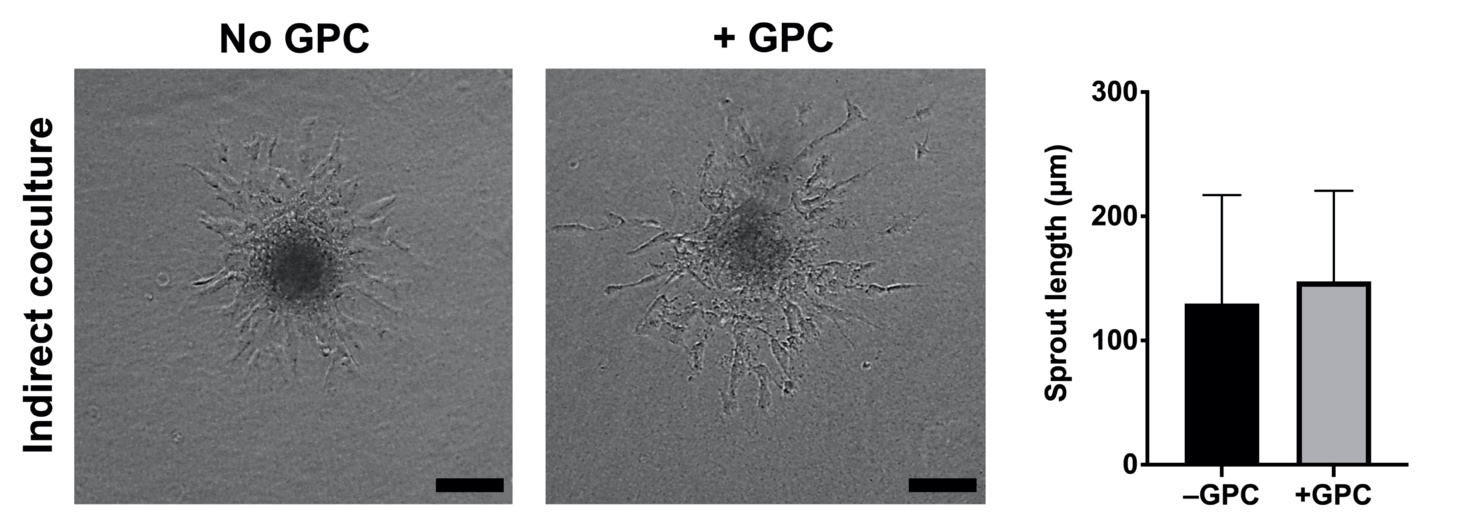
**

Representative images of HUVEC-sprouting in the absence (No/– GPC) and presence of monolayer GPC (+GPC) after 72 h (scale bars 100 μm) and quantification of corresponding sprout lengths; data represent means + SD of at least 3 experimental repeats.

- 1. **Supplementary figure 2: Coculture spheroids**

**A**

**
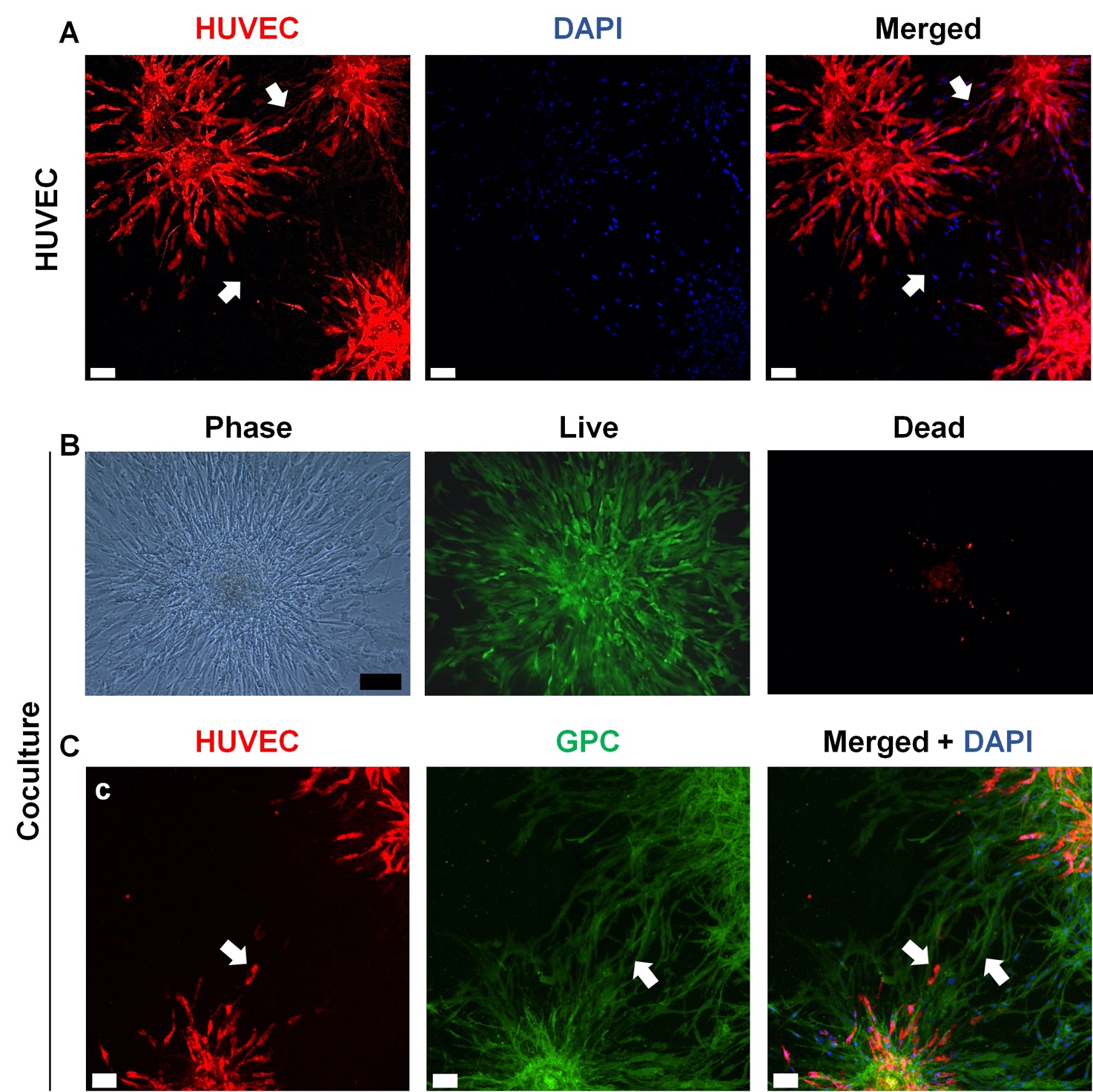
**

**B**

AA

AA

(A) Representative phase and fluorescent microscopy images of cell viability in coculture spheroids; scale bars 100 µm. (B) Representative confocal images showing sprout formation in HUVEC-GPC coculture spheroids; GPC migration preceded HUVEC sprouting and provided a substrate for sprout elongation (white arrows); scale bars 50 µm. HUVEC and GPC are stained with red and green cell-labelling dyes, respectively; nuclei are stained with DAPI.

- 1. **Supplementary figure 3: Optimization of HPLG**


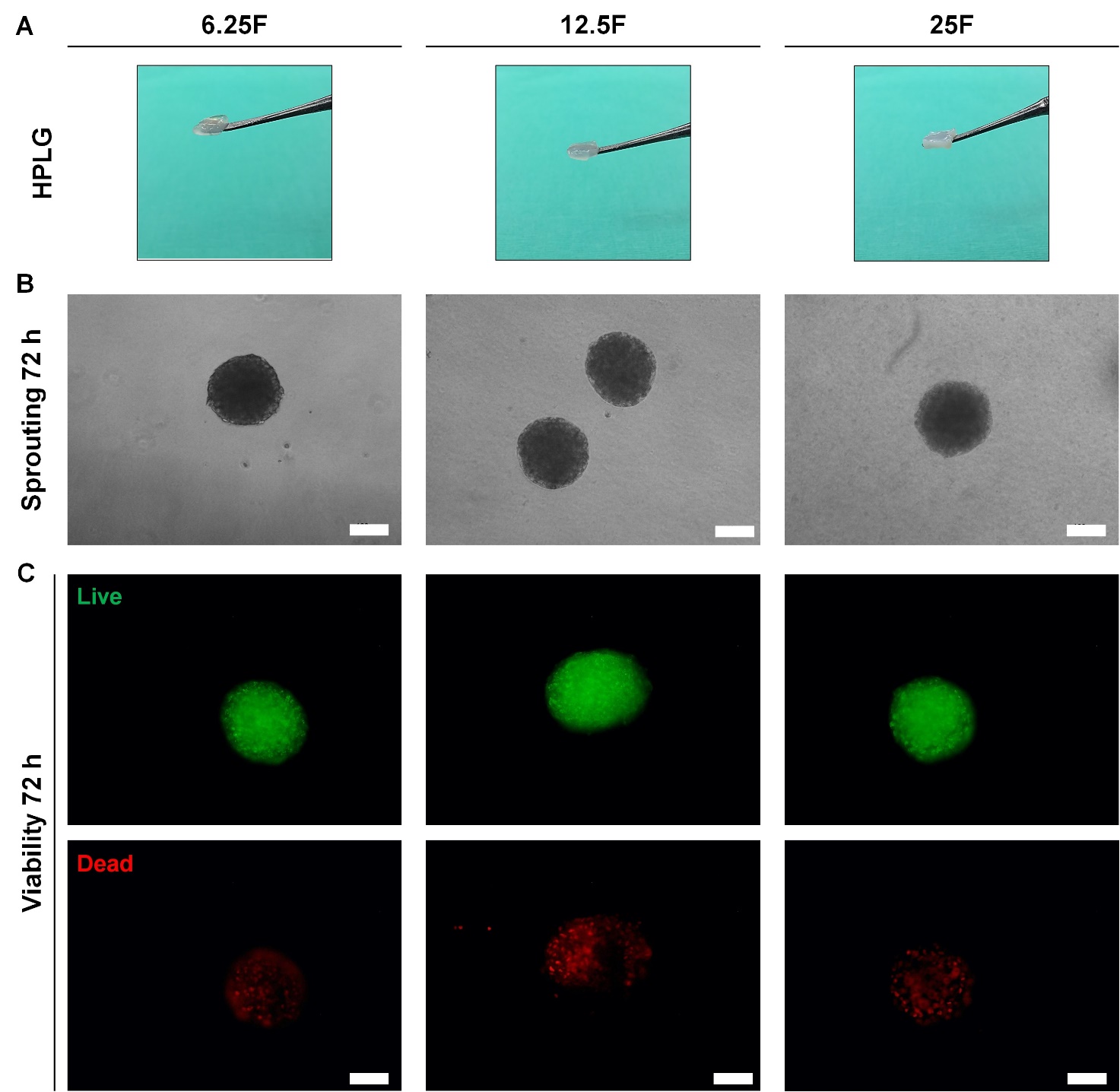


(A) Representative photographs modified HPLG supplemented with 6.25 (6.25F), 12.5 (12.5F) or 25 (25F) mg/mL fibrinogen. (B) Representative images of HUVEC spheroids in the corresponding HPLG – note the absence of sprouting; scale bars 100 µm. (C) Cell viability via LIVE/DEAD staining in the corresponding HPLG after 72 h; scale bars 100 µm.

- 1. **Supplementary figure 4: Rheology of HPLG**

**
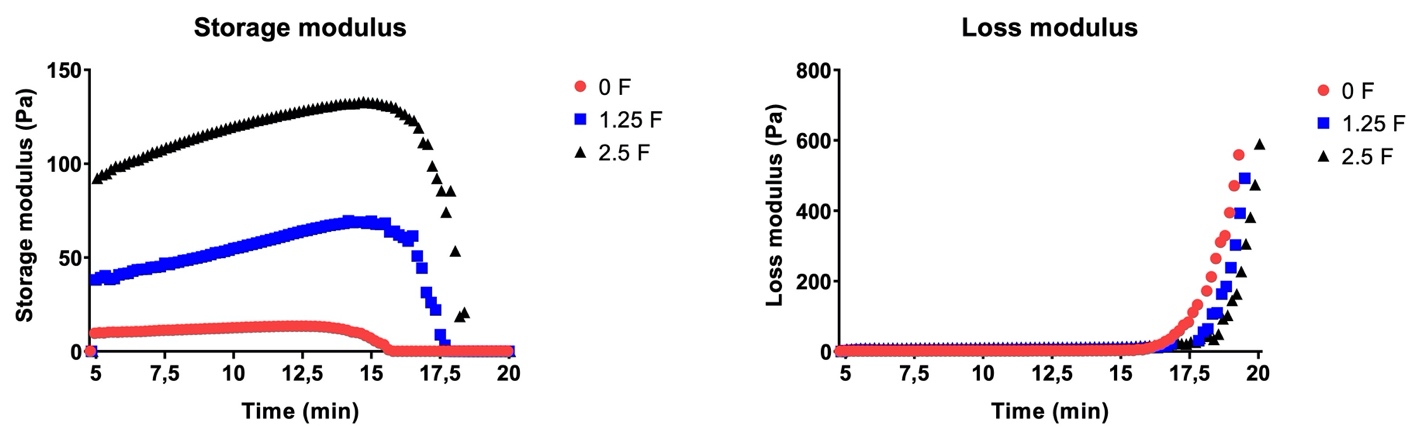
**

Rheological analysis indicating storage modulus and loss modulus of 0F, 1.25F and 2.5F HPLG.

- 1. **Supplementary figure 5: Confirmation of sprouting in CAM-implanted spheroids**


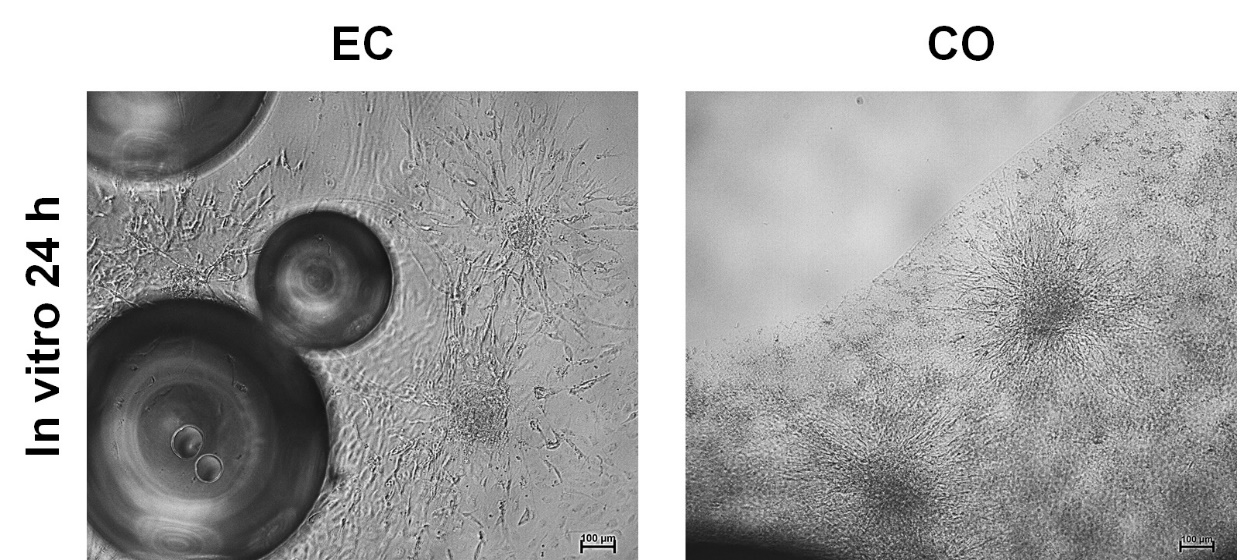


Sprouting of HUVEC (EC) and coculture (CO) spheroids in HPLG, seeded in parallel to the *in vivo* CAM assay; scale bars 100 μm.
